# Functional and Structural Architectures of Allocentric and Egocentric Spatial Coding in Aging: A Combined DTI and fMRI Study

**DOI:** 10.3389/fneur.2021.802975

**Published:** 2022-01-28

**Authors:** Abiot Y. Derbie, Bolton K. H. Chau, Chetwyn C. H. Chan

**Affiliations:** ^1^Applied Cognitive Neuroscience Laboratory, Department of Rehabilitation Sciences, The Hong Kong Polytechnic University, Kowloon, Hong Kong SAR, China; ^2^Department of Psychology, Bahir Dar University, Bahir Dar, Ethiopia; ^3^Department of Psychology, The Education University of Hong Kong, Tai Po, Hong Kong SAR, China

**Keywords:** FPAN, allocentric spatial coding, egocentric spatial coding, spatial representation, frame of reference, white matter integrity, functional magnetic brain imaging (fMRI)

## Abstract

**Background:**

Aging disrupts the optimal balance between neural nodes underlying orienting and attention control functions. Previous studies have suggested that age-related changes in cognitive process are associated to the changes in the myelinated fiber bundles, which affected the speed and actions of the signal propagation across different neural networks. However, whether the age-related difference in allocentric and egocentric spatial coding is accounted by the difference in white-matter integrity is unclear. In this study, using diffusion tensor imaging (DTI) and functional magnetic resonance imaging (fMRI), we sought to elucidate whether age-related differences in white matter integrity accounts for the difference in nodes to the distributed spatial coding-relevant brain networks.

**Material and Method:**

Older (*n* = 24) and younger (*n* = 27) participants completed the structural DTI and fMRI scans during which they engaged in a cue-to-target task to elicit allocentric or egocentric processes.

**Results and Conclusion:**

Efficient modulation of both allocentric and egocentric spatial coding in fronto-parietal attention network (FPAN) requires structure–function interaction. Allocentric task-modulated connectivity of the fronto-parietal network (FPN) and dorsal attention network (DAN) with the temporal lobe was influenced by the aging differences of the white-matter tracts of the posterior and superior corona radiata (PCR and SCR), respectively. On the other hand, aging difference of the superior longitudinal fasciculus mainly influenced the egocentric-task-modulated connections of the DAN and FPN with frontal regions and posterior cingulate cortex. This study suggested that functional connections of the FPAN with near and far task-relevant nodes vary significantly with age and conditions.

## Introduction

Functional neuroimaging studies demonstrated that the neural substrates mediating allocentric and egocentric spatial coding (aSC and eSC, respectively) are dissociable (1, 2). Age-related differences in the working mechanisms of aSC and eSC have also been observed [for recent review: (3)]. On the other hand, with the absence of any significant disease, aging is characterized by the degeneration of white matter integrity, demyelination, and axonal loss. Alternations of the white matter integrity have been found to alter the transmission of the visuospatial neural signals to the near and far brain regions for information processing (4–6). However, how these age-related alternations would influence the spatial coding corresponding functional networks, and hence the dissociation between the aSC and eSC has not been explored.

Visuospatial attention is subserved by the functional interactions within the fronto-parietal attention network (FPAN) (2, 7, 8). The two subregions, namely the intraparietal sulcus (IPS) and the premotor cortex (PMC) including the frontal eye-fields (FEF) (1, 8) are involved in sensorimotor integration and spatial relationships among objects in space (9–11). The FPAN's subregions of the posterior parietal cortex (PPC) and lateral prefrontal cortex (LPFC) (2, 12) are involved in encoding context dependent and trial-by-trial modulation of attention (such as shifts and reorienting of attention) and response inhibition (10, 13). Common neural substrates have been found between the aSC and eSC along the FPAN. For instance, Szczepanski et al. (2) used a cue-to-target paradigm to elicit the neural related processes of aSC and eSC. The results indicate the supplementary eye-field (SEF) to superior parietal lobule (SPL) as the neural pathway common associated with both the eSC and aSC. The neural pathway of FEF to intraparietal sulcus area two (IPS2) was unique to the eSC. Other studies revealed that aSC tends to demand working memory resources, which involved the MTL (14–16).

The connectivity between the PPC and LPFC is found to be involved in attentional control (7, 17). The fiber tract of the superior longitudinal fasciculus (SLF) has been identified to strengthen the functional connectivity between the near and far neural nodes of the PPC and LPFC (18). Previous studies indicated that functional connectivity within the core nodes of the FPAN influenced participants' reaction times (RT) on visuospatial task (19). In addition, the fractional anisotropy (FA) of the SLF was correlated with the neural activities in the FPAN during visuospatial attention (20). Other correlations between the RTs and the FA values were in the splenium of the corpus callosum (SPN) (5), right posterior thalamic radiation (PTR) (21), bilateral inferior longitudinal fasciculus (ILF) (4), anterior corona radiata (ACR) (22), and posterior corona radiata (PCR) (23). It is noteworthy that the RT–FA relationships are more prominent in the right than the left hemisphere, which is in-line with the right-hemispheric dominance in visuospatial attention (24). Taken together, visuospatial attention is subserved by the FPAN, which involves structure–function interactions.

The intra-parietal lobule (IPL) is a major structural hub with fiber tracts passing through the inferior and middle longitudinal fasciculus (ILF and MLF) (25). The ILF connects the IPL with the middle temporal gyrus (MTG), inferior temporal gyrus (ITG), and superior occipital gyrus (SOG). The inferior occipitofrontal fascicle (IOF) connects the IPL with the precuneus and superior frontal gyrus *via* the caudate, and the SLF connects the IPL with the middle frontal gyrus (MFG) and inferior frontal gyrus (IFG) (25). The left and right IPL are connected *via* the splenium of the corpus callosum (SPN) (26). Disruption of these structural connectivities has been shown to affect the underlying functional mechanisms of aSC and eSC. Complimentary evidence from lesion studies have shown that disruption of the connection of SLF, ILF, and inferior fronto-occipital fasciculus (IFOF) disrupted neural activities of the middle frontal gyrus (MFG), supramarginal gyrus (SMG), and postcentral gyrus (PoCG) during eSC and neural activities of the superior temporal (ST), middle temporal (MT), angular gyrus (AG), and middle occipital gyrus (MOG) during aSC (18, 20, 27).

On the other hand, the caudal part of the IPL (cIPL, known as angular gyrus) projects the signals received to the parieto-premotor and parieto-medial temporal pathways (1). The parieto-premotor pathway is involved in eSC, and its core neural substrates are the cIPL, superior parietal lobule (SPL; including IPS), somatosensory motor area (SMA), and FEF (1, 28, 29). IPS is related to attention and FEF is related to the action template formations (7, 18, 30). The parieto-medial temporal pathway, on the other hand, is involved in aSC (1), and its key neural substrates are the caudal part of IPL (area PG) (31, 32), PCC (33), the retrosplenial cortex (RSC), temproparietal junction (TPJ), and medial temporal lobule (MTL) (34, 35). With strong connections to the PCC and TPJ, the information received by the cIPL is transformed to an aSC representation mediated by the precuneus (14). The precuneus is related to spatial updating (12) and the PCC is related to shifting spatial attention (1, 36). These studies highlighted the important common and distinct roles played by the IPL-related functional and structural connectivity networks, in particular the cIPL, in the eSC and aSC.

Aging disrupts the optimal balance between neural nodes underlying visual attention along the FPAN (34), such as alerting (37), orienting (38), and attention control (39). Such disruption was more prominent in the dorsal parts of FPAN, expressed in decline top–down attentional guidance (5). Age-related changes in orienting attention was associated with the disruption of WM integrity in the SLF and ILF (17). The disrupted WM integrity has been associated to slow down RT among older adults (4). The SLF and ILF fiber tracts influencing the PFC subserve to attentional control, whereas that influencing the PPC subserves orienting attention (40). The WM integrity in the ACR was found related to reduced attentional control in older adults (41) and lowered neural activities in the MT FEF and LPFC (42). Specific to spatial coding, older adults were reported to tend to prefer the egocentric (43) than allocentric orienting (44). Such preference was suggested due to the reduction in functional connectivity between PFC and the parietal regions (40). Subsequently, the eSC to aSC preference is further explained by the latter demands, more visual short-term memory than the former (45). Aging was also suggested to affect the pathway of the PCC (40) and SPL to the LPFC *via* the MT (1, 14, 46), which subserves the retrieval strategy and transformation of visual representation for forming a mental map in aSC [recent review and metaanalysis: (3, 30)]. The alterations of the structure–function relationships along the SPL, PCC, MTL, and LPFC may lead to the age-related changes in the aSC but not in the eSC.

Previous lesion studies revealed atrophies to the fiber bundles in the SLF, ILF, and IFOF altered the functional connectivity within the FPAN, affecting both the aSC and eSC functions (18, 20, 27). Yet, the underlying mechanism is not well understood. In the present study, we combined structural MRI (diffusion tensor imaging, DTI) and event-related fMRI to investigate how changes in the FPAN's white-mater integrity and brain activations due to old age can explain the unique age-related decline in aSC but not in eSC.

We hypothesized that the aSC task-specific effective connectivities between the fronto-parietal network (FPN) (PPC and LPFC) and the DAN (IPS and FEF) would significantly associate with the FA values of the SLF, PCR, and SCR fiber bundles for both the older and younger groups. In contrast, the eSC task-specific effective connectivities within the FPAN (involving IPS, FEF, PPC, and LPFC) would not significantly associate with the FA values of the SLF, PCR, and SCR tracts.

## Methods

### Participants

A total of 51 volunteers participated in the study. Among them were 27 younger (Mean: 22.37 years, SD = 0.88, 18 women) and 24 were older adults (Mean: 68.29 years, SD = 3.59, 13 women). All participants were right-handed and had normal or corrected-to-normal vision. They had normal cognitive functions as screened by the Chinese version of Montreal Cognitive Assessment [MoCA; (47)]. The MoCA scores were not significantly different between the younger and older groups (Table 1). The MoCA has been shown to be a reliable measure of cognitive functions in spatial memory (48), attention, and executive functions (49) in aging studies. Ethical approval was obtained from the ethic committees of the Affiliated Rehabilitation Hospital, Fujian University of Traditional Chinese Medicine, where the study was conducted. All participants gave written informed consent prior to engaging in the testing and brain imaging experiment.

**Table 1:** Demographic characteristics of the participants.

	**Younger adults**	**Older adults**
	**(*N* = 27)**	**(*N* = 24)**
Age	22.37 ± 0.88	68.29 ± 3.59
Sex (m/f)	9/18	11/13
Years in school	14.59 ± 0.50	11.33 ± 2.88
MoCA score	28.25 ± 1.43	26.21 ± 1.68

#### Apparatus and Stimuli

Stimuli were displayed on a 30-inch MR-compatible LCD monitor that was placed outside of the MRI brain scanner bore above the head of the participant. Participants viewed the stimuli through a mirror attached to the head coil.

#### Cueing Paradigm and Procedures

**Figure 1:**
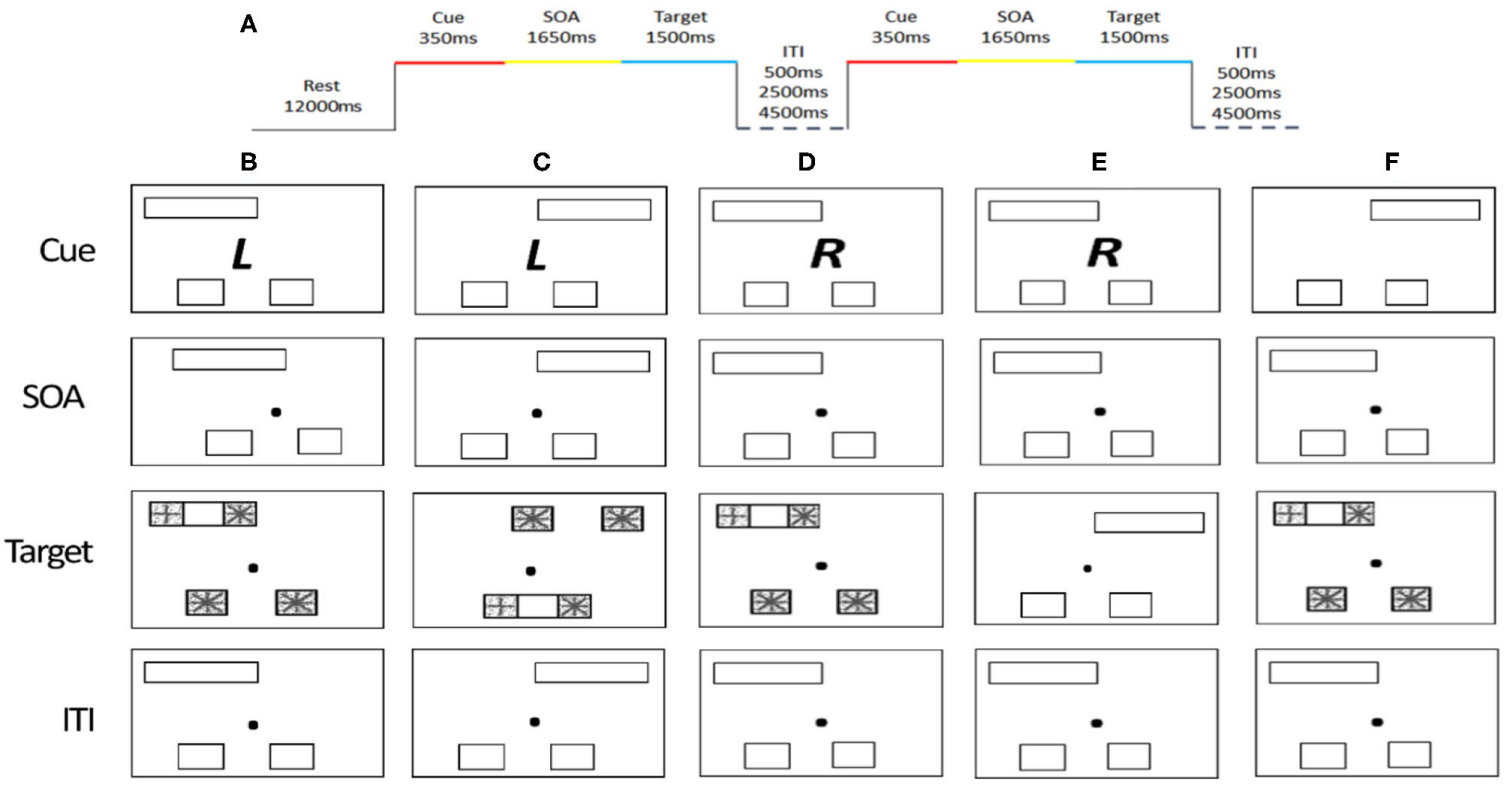
Cue-to-target paradigm and timing parameters. **(A)** Stimulus timing. **(B)** Valid trial for the allocentric condition. **(C)** Valid trial for the egocentric condition. **(D)** Invalid allocentric trial, where the cue does not carry valid information about the probable location of the target stimuli. **(E)** Cue-only trial, where the cue is not followed by a target. **(F)** Neutral trial, where the cue carries no important information. The cue (RandL) is enlarged and the stimuli are displayed here in reverse contrast but appear in a white figure on a black background.

The task used in the fMRI scan involved detection of shapes with a cue-to-target paradigm adapted from Wilson, Woldorff, and Mangun (50). The shape detection task has been used to study attention control networks. Each trial began with the presentation of three stimuli and a cue for 350 ms (Figure 1). The three stimuli included a pair of empty squares (subtended 3.75° vertically and horizontally to the center) and one empty rectangle (displayed at 3.75° vertically and 12.2° horizontally to the center). The cue was a Chinese character presented at the center that indicates the type of response required later in the trial. The cue was followed by a stimulus-onset asynchrony (SOA) for a fixed duration of 1,650 ms, in which the Chinese character was replaced by a dot. Next, a new pair of squares appeared inside the two ends of the empty rectangle, i.e., a total of four squares on the screen. One of the four squares showed a “plus” sign that indicates a target and the other three show “asterisks” that indicates distracters. In the eSC condition, the target would appear in one of the squares outside the rectangle and participants had to indicate whether the target was located on the left or right according to their own bodily coordinates by pressing a button using middle or index finger, respectively. In the aSC condition, the target would appear in one of the squares inside the rectangle, and participants had to indicate whether the target was located on the left or right side of the rectangle, regardless of their own bodily coordinates. In the cue phase, at the beginning of the trial, the Chinese character cues the identity of the trial and the position of the target. The font is in italic or a normal format that indicates an egocentric or allocentric type of response, respectively. The character is either 左 (left) or 右 (right) indicating the target would appear on the left or right respectively in its corresponding condition. Finally, an intertrial interval with a varying delay of 500, 2,500, or 4,500 ms is organized in a random order. The combinations of the words and the fonts of the words were counterbalanced across all the participants.

There are four types of trials: valid, invalid, neutral, and cue-only. On the valid trials, the location of the target is consistent with the information provided by the cue. On invalid trials, the location of the target is inconsistent with the information provided by the cue. On cue-only trials, it does not include any target and distracters; rather a dot at the middle of the screen was presented. On the neutral trials, the cue provides no information of the location of the target. A total of 288 trials (144 valid, 48 invalid, 72 cue only, and 24 neutral trials) were presented across three runs, containing 96 trials per run. The distribution of each trial type to the left or right was counter balanced. It took about 30 min to complete all the three runs.

## Training Session

All participants had to complete a training session to get familiarize with the task before the actual experiment. The trials used for the training and actual experiment were similar, except that in the training session each response was followed by a feedback. All participants were to achieve an accuracy rate of at least 80% of the total trials before engaging in the experiment.

### Functional MRI and DTI Image Acquisition and Preprocessing

#### Image Acquisition and Scanning Parameters

Siemens Prisma 3.0 T MRI system (Germany) with a 64-channel coil was used for the image data acquisition. High-resolution structural T1-weighted images were acquired: echo time (TE) = 2.27 ms, repetition time (TR) = 2,300 ms, field of view (FOV) = 250 × 250 × 240 mm^3^, voxel size = 0.98 × 0.98 × 1 mm^3^, slice thickness = 1.0 mm; image matrix = 256 × 256). Functional images were acquired using a T2-weighted echo planar imaging (EPI) sequence: 37 noncontiguous slices of gradient-echo EPI with TE = 30 ms; TR = 2,000 ms; field of view (FOV) = 230 × 230 × 146 mm^3^; voxel size = 3.6 × 3.6 × 3.6 mm^3^; slice thickness = 3.6 mm; slice gap = 0.36 mm; image matrix= 64 × 64. Diffusion-weighted spin-echo planar images for diffusion tensor imaging (DTI) were obtained: TR = 5,000 ms, TE = 69 ms; flip angle, 90°; matrix, 96 × 96; 35 sagittal slices with thickness 3.5 mm; FOV = 224 mm; bandwidth = 1,954 Hz/voxel; voxel size = 1.8 × 1.8 × 3.5 mm^3^. Diffusion-weighting gradients were applied at a b value of 1,000 s/mm^2^. Twelve images with no diffusion gradients (b0) was also acquired for each participant.

### Functional MRI Preprocessing and Univariate Analysis

#### Functional MRI Preprocessing

Preprocessing of the event-related fMRI BOLD signals of the participants was carried out by using FSL version 6.0.0 (FMRIB Software Library; University of Oxford; www.fmrib.ox.ac.uk/fsl) (51). The preprocessing included the removal of non-brain structure using brain extraction tool [BET; (52)], motion correction using MCFLIRT (53), temporal high-pass filtering with 100 s cut-off, slice timing correction, spatial smoothing by a Gaussian kernel with full-width half-maximum of 8 mm. Functional images were, then, registered to its native anatomical image using FMRIB's linear image registration tool (FLIRT) and then linearly registered to the MNI152 high resolution T1 2 mm template brain with 12 degree of freedom affine transformation (53, 54). To allow for signal stabilization, the first two dummy scans of each run were discarded.

#### Diffusion-Weighted Image Processing

The DTI data of the participants were analyzed using the FMRIB Software Library. The image with no diffusion gradients (b0) from each subject was skull-stripped using FSL's brain extraction tool (52). All diffusion weighted data from all subjects were preprocessed for eddy-current induced distortions and motion correction using the FSL's topup and Eddy tool. After distortions and motion correction, using the FDT toolbox (51), raw DTI data was fit into the diffusion tensor model from which the FA (fractional anisotropy) maps for each participant was generated.

#### Tract-Based Spatial Statistics

**Figure 2:**
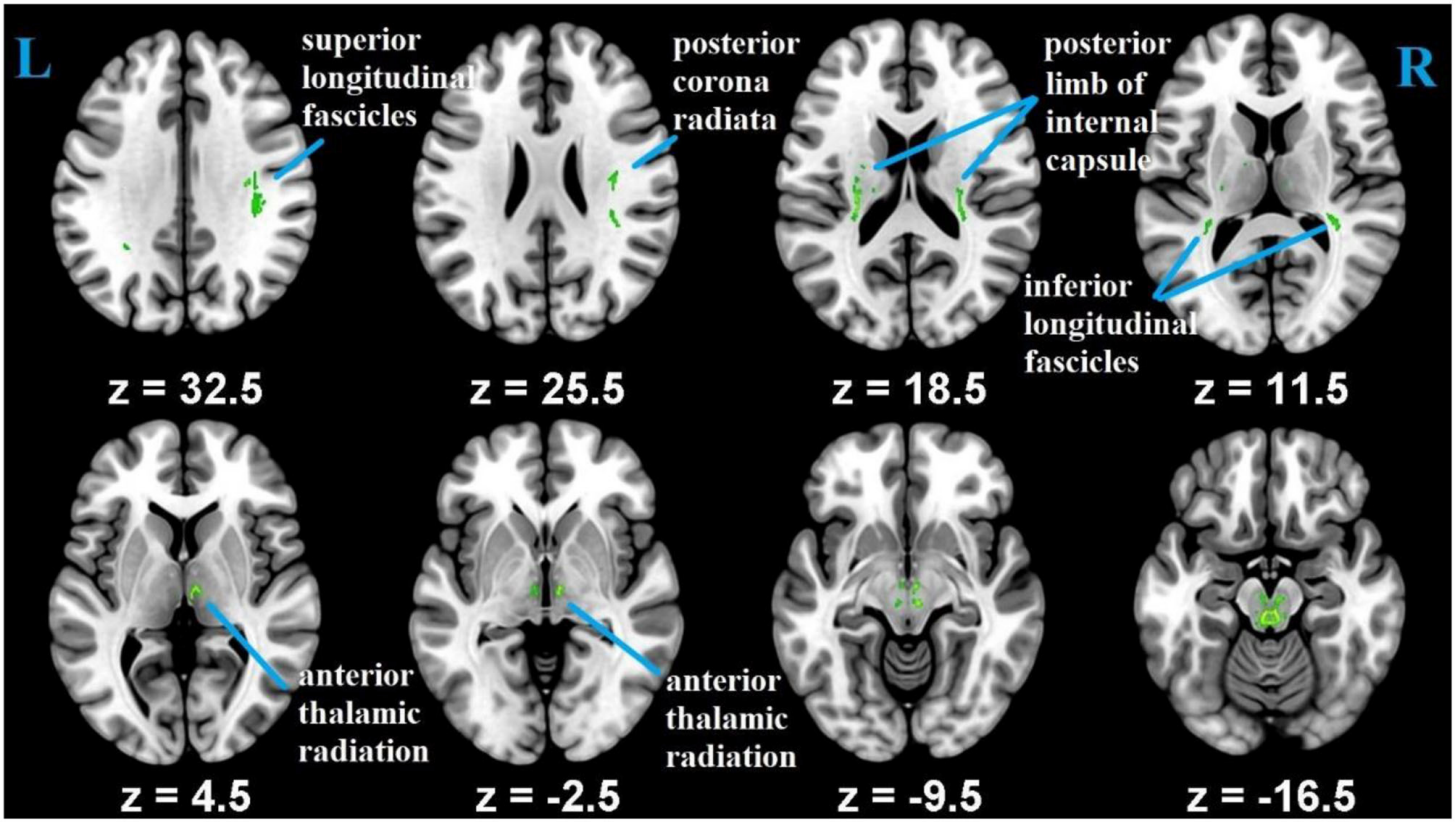
Differences in FA measures between the younger and older groups.

Whole-brain voxel-wise statistical analysis was carried out with the tract-based spatial statistics [TBSS; (55)] parts of the FSL (51). First, all of the participants' FA images were aligned into FMRIB58_FA 1 × 1 × 1 mm standard-space using FNIRT (FMRIB's Non-linear Registration Tool) (56). Second, to achieve skeletization, the aligned FA images were then affine-transformed into 1 x 1 x 1 mm^3^ MNI152 space. Third, using the mean FA image, FA skeleton common to crosssubject and crossgroup white-matter tracts was created. This was achieved by thresholding the center of white-matter bundles with a value of 0.2. Each subject's aligned FA maps were then projected onto the mean FA skeletonized map, and the resulting data was subjected for crossgroup voxel-wise statistics. Correction for multiple testing was conducted using threshold-free cluster enhancement (TFCE) method (57) and determined at *p* ≤ 0.05. As an additional quality assurance, we tested the difference in FA between young and old, and the results were consistent with previous studies that reported aging difference (4) (Figure 2). The mean FA values were, then, extracted from each participant using predefined ROI's as a mask as explained below.

#### Creation of Functional Networks and White-Matter Region of Interests (ROIs)

**Figure 3:**
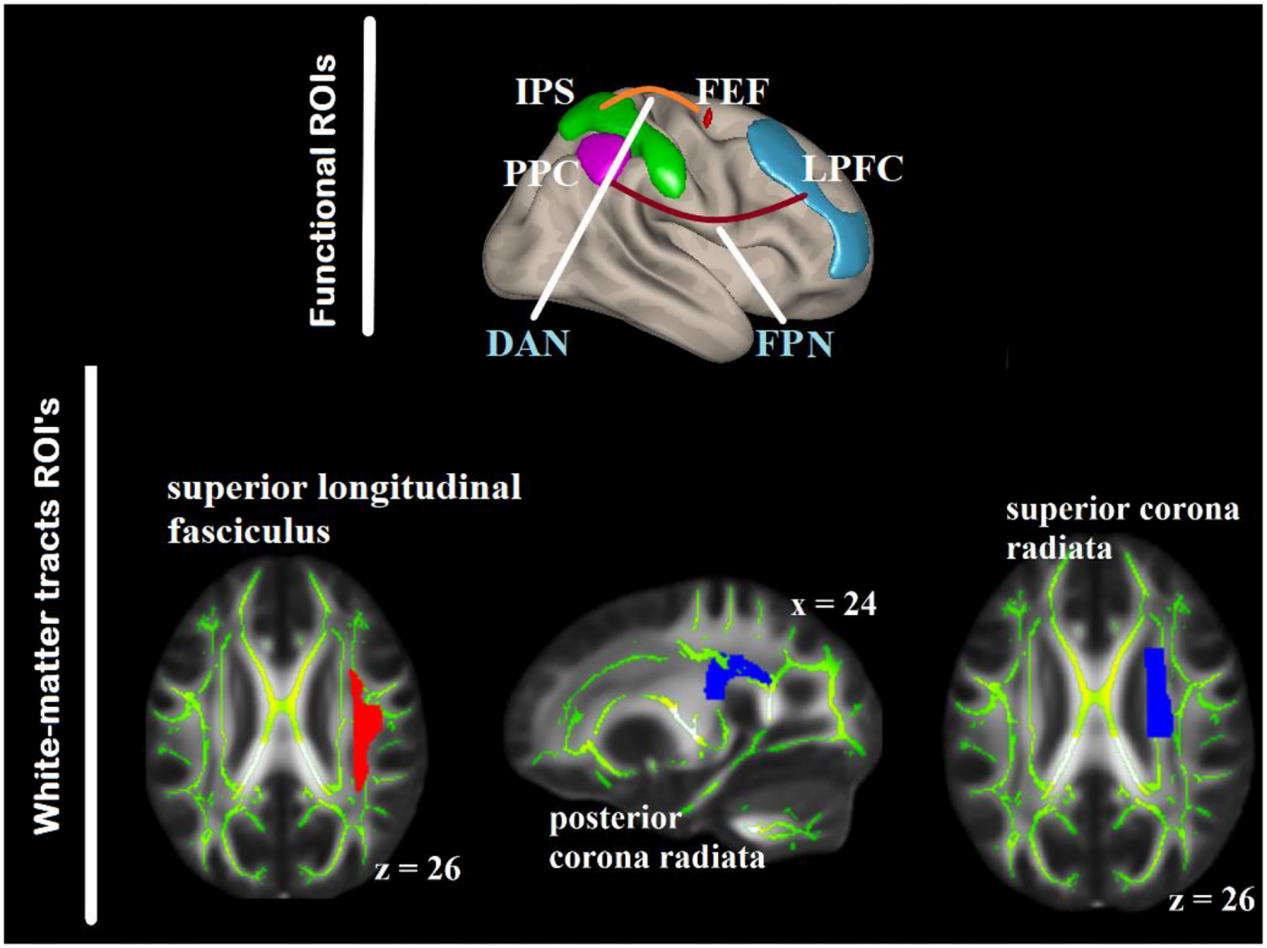
ROIs within the DAN and FPN as seed regions for the gPPI analysis (**top**) defined based on anatomical areas from the Harvard–Oxford atlas included in CONN. The white-matter ROIs are overlaid on the FMRIB58_FA_1-mm standard-space and the mean FA skeleton of both the groups (**bottom**). DAN, dorsal attention networks; FPN, fronto-parietal network; PPC, posterior parietal cortex; IPS, intra-parietal sulcus; FEF, frontal eye-fields, LPFC, lateral prefrontal cortex.

To delimit the analysis and reduce the type I error, given the number of voxel to ROI comparisons, two key networks associated with visual attention and top–down attention control, i.e., DAN and FPN were included for the labeling of the functional ROIs according to the anatomical areas derived from the Harvard–Oxford atlas of the CONN (58, 59) (Figure 3).

There were two steps for defining the white-matter ROIs. First, we identified published studies on white-matter (particularly FA) in relation with aging and visuospatial attention. Ten key white-matter tracts were selected from five studies (4, 5, 21, 60, 61). The white-matter tracts identified were the anterior and superior corona radiata (SCR) bilaterally, PCR bilaterally, body corpus callosum (BCC), splenium corpus callosum (SPN), SLF bilaterally, and posterior thalamic radiation (PTR) bilaterally. WM labeling and parcellation was done by using the FSL atlas tools provided by Johns Hopkins University [“JHU ICBM-DTI-81”; (62)]. Second, we correlated the FA values and RT of the present data. Three white-matter tracts were correlated in one or more of the RT in older group or younger group in either aSC or eSC condition, and their contributions to the variation of the task-relevant BOLD signal were above and beyond the other white matter tracts. Hierarchical multiple regression was used to test whether one white-matter tract contribution to the variation in the task-relevant BOLD signal is above and beyond the other white-matter tract. They were the right SCR, right PCR, BCC, SPN, and right SLF (Figure 3). These tracts were binarized, and used as a mask to interrogate the FA values from each participant using “fslmeants” on the all_FA_skeletonized image in FSL. After obtaining the functional and structural ROIs, we tested the relationships between the aSC and eSC task-related effective connectivities within the FPN (cIPL and LPFC) and DAN (IPS and FEF) with the FA values in the PCR, SLF, and SCR. To achieve this, the model for the seed-to-voxel effective functional connectivity analysis contains young FA > old FA as in the between-subject contrasts, aSC > eSC as in the between-condition contrasts, and one seed region in each of the DAN and FPN as in the between source contrasts.

#### Seed-to-Voxel Connectivity Analysis: Generalized Psycho-Physiological Interaction

The association between functional connectivity and FA during allocentric and egocentric spatial condition along the FPAN was examined using the seed-to-voxel effective functional connectivity analysis of the CONN toolbox (63), following gPPI (64). Using gPPI, we extracted the average BOLD time series from four predefined seed region masks. In our data, both the FA with RT and FA with BOLD relationships were lateralized to the right hemisphere. Studies have also shown that visuospatial attention is mainly maintained by the right hemisphere (1, 65), and thus only the right FPAN seed regions were drawn. The regions were the FEF, IPS, and LPFC. IPS and FEF are parts of the DAN which has been found to relate to object in space (7, 18) and PPC and LPFC are parts the FPN which are related to attentional control (7, 8). The following gPPI regressors were modeled:

1. All of the task effect (allocentric, egocentric, and neutral) convolved with hemodynamic response function (HRF);
2. The seeds (IPS, FEF, LPFC, and PPC) BOLD time series with a task regressors corresponding to the allocentric, egocentric, and neutral; and,
3. The interaction term of those seed regions time series with a task regressor corresponding to the three conditions convolved with HRF.

The aSC, eSC, and neutral-specific connectivity regressors were submitted to a gPPI model to conduct task-modulated seed-to-voxel connectivity analyses. Each seed-to-voxel gPPI map reflecting the regressors above were constructed for each participant. The seed-to-voxel gPPI maps were used to test the effects of between-subject and between-condition contrasts at the group level across each seed.

The gPPI contrast maps for each model were generated, and the results were displayed using the statistical parametric mapping (SPM12). The corresponding group-level beta-weights for each contrast were extracted and plotted along the connectivity brain maps.

## Results

### The Association Between Functional Connectivity and FA in Aging During Allocentric and Egocentric Spatial Coding

**Figure 4:**
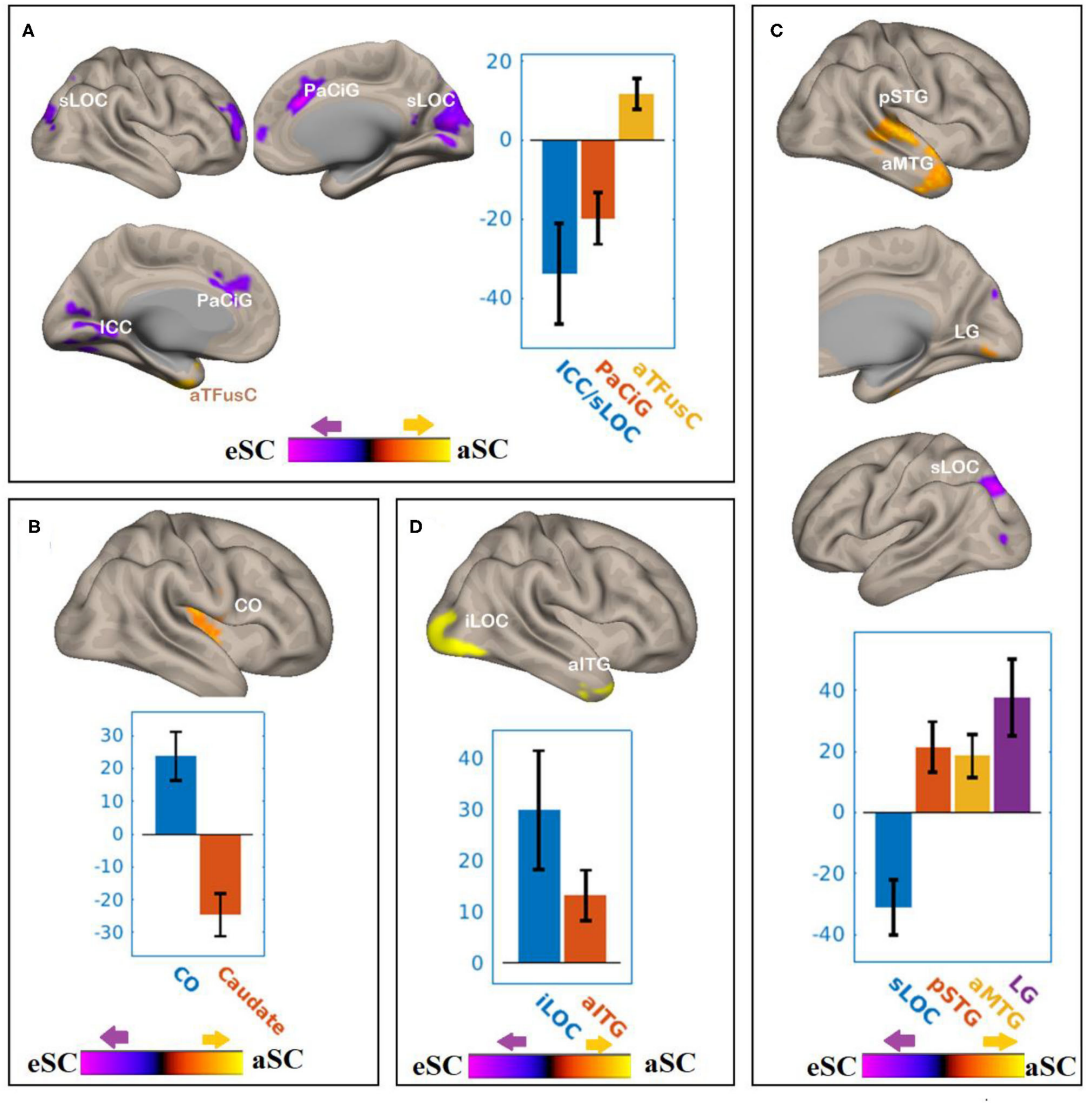
The differences between the younger and older participants in association between the FPN and DAN connectivity the with the FA values of PCR under the influence of the allocentric and egocentric task effects. **(A)** PCR–FEF for aSC > eSC. **(B)** PCR–IPS for aSC > eSC. **(C)** PCR–LPFC for aSC > eSC. **(D)** PCR–PPC for aSC > eSC. The connectivity blobs are thresholded and cluster-level corrected using FDR for multiple comparison at *p* = 0.05. Color bar coding: Violet represents an increased (positive) and reduced (negative) in functional connectivity for the eSC and aSC condition, respectively, whereas the hot represents an increased (positive) and reduced (negative) in functional connectivity for the aSC and eSC condition, respectively. sLOC, superior division of lateral occipital cortex; ICC, intracalcarine cortex; PaCiG, Para-cingulate gyrus; CO, central opercular cortex; pSTG, posterior division of superior temporal gyrus; aMTG, anterior division of middle temporal gyrus; LG, lingual gyrus; iLOC, inferior division of lateral occipital cortex.

There were associations between FA in the PCR and DAN under the influence of the aSC and eSC task effects (Figure 4, Table 2). Compared to the older participants, FA values of the PCR for the younger participants showed significant positive association with the connectivity between the right FEF and the anterior temporal fusiform cortex (aTFusC) [T(47) = 5.05, *p* < 0.01] and showed negative association with the connectivity between the right FEF and superior division of lateral occipital cortex (sLOC) [T(47) = −4.62, *p* < 0.001] and between the right FEF and PaCiG [T(47) = −5.18, *p* < 0.001] in the aSC > eSC contrast. Moreover, for the aSC condition, the younger participants showed significant association between connectivity in the right IPS and central opercular cortex [CO: T(47) = 5.33, *p* < 0.03] and negative association with connectivity between the right IPS and the caudate [T(47) = −6.39, *p* < 0.03].

**Table 2:** Summary on the results based on the seeds constructed within the DAN and FPN for the young_PCR > old_PCR as the between-subject contrast and aSC > eSC as the between-condition contrast.

**Seed**	**Functional connectivity** **region**	**K_E_**	**Hemi**	**Coordinates**			***P*_FDR_**	**Peak** **t**
				***X***	***y***	***Z***		
FEF	Superior lateral occipital cortex	2,254	L	−18	−66	2	<0.001	−4.62
	Paracingulate gyrus	1,492	R	10	28	28	<0.001	−5.18
	Anterior temporal fusiform cortex	650	L	−30	−4	−40	0.01	5.05
IPS	Central opercular cortex	843	R	42	−18	20	0.03	5.33
	Caudate	832	R	2	−20	14	0.03	−6.39
LPFC	Lingual gyrus	885	R	30	−100	−8	0.02	5.73
	Posterior superior temporal gyrus	711	R	62	−20	0	0.03	4.25
	Anterior middle temporal gyrus	680	R	56	14	−32	0.03	4.34
	Superior lateral occipital gyrus	630	L	−18	−78	−40	0.03	−5.77
PPC	Inferior lateral occipital cortex	994	R	38	−84	−10	0.02	4.34
	Anterior inferior temporal gyrus	786	R	36	16	−44	0.04	4.47

There were associations between the FA values in the PCR and FPN under the influence of the aSC and eSC task effects (Figure 4, Table 2). Compared to the older participants, the FA values of the PCR among the younger participants were significantly associated with the connectivity between the right LPFC and LG [T(47) = 5.73, *p* < 0.02], right LPFC and pSTG [T(47) = 4.25, *p* < 0.03], right LPFC and aMTG [T(47) = 4.34, *p* < 0.03], and significantly but negatively associated with the connectivity between the right LPFC and sLOC [T(47) = −5.77, *p* < 0.03] in the aSC > eSC contrast. Moreover, for the aSC condition, the FA values of the PCR for the younger participants showed significant association with the connectivity between the right PPC and iLOC [T(47) = 4.34, *p* < 0.02] and between right PPC aITG T(47) = 4.47, *p* < 0.04).

**Figure 5:**
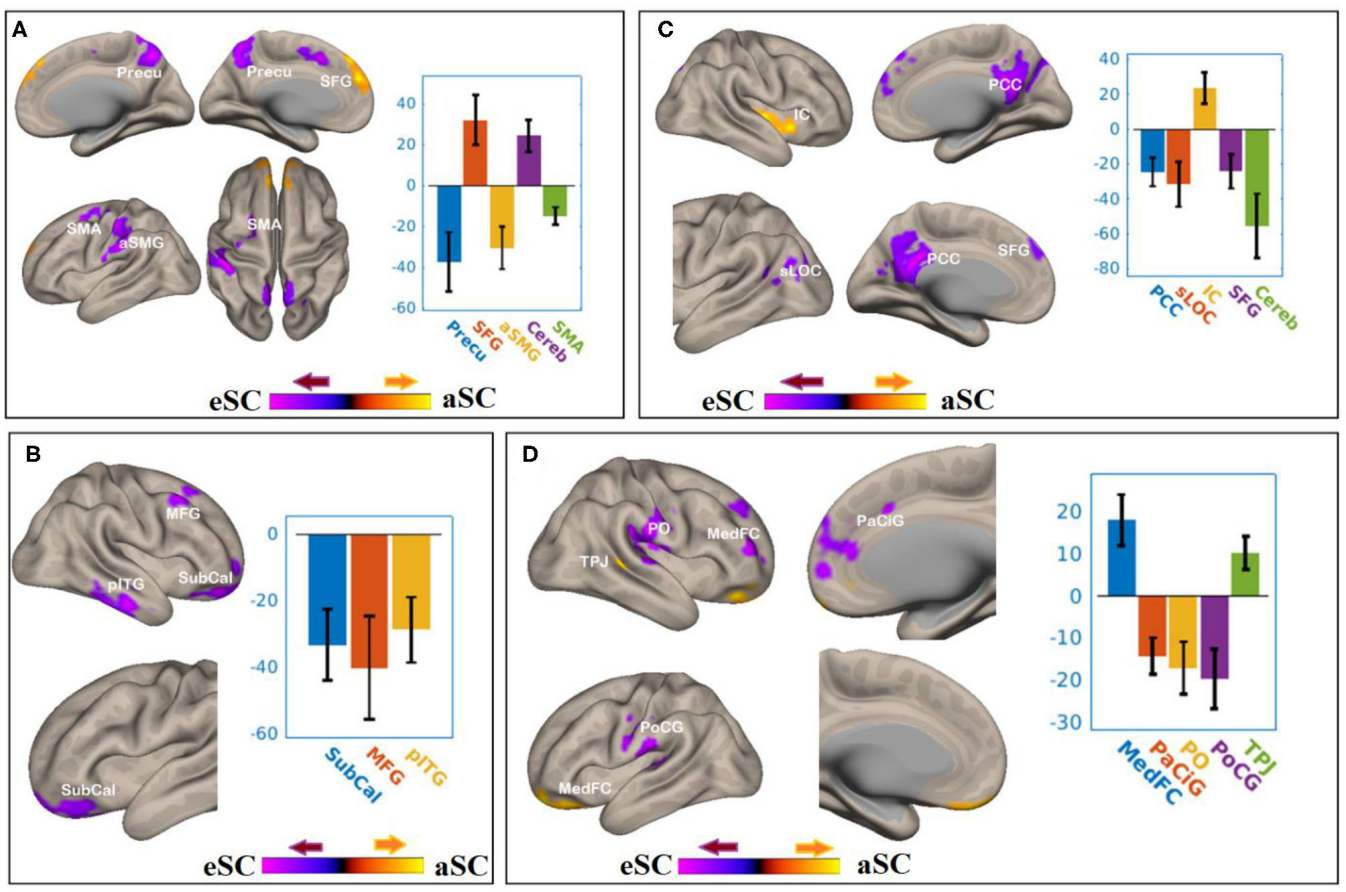
The difference between the younger and older participants in the FPN and DAN connectivity in association ith the FA values of the SLF under the aSC and eSC task effects. **(A)** SLF–FEF for aSC > eSC. **(B)** SLF–IPS for aSC > eSC. **(C)** SLF–LPFC for aSC > eSC. **(D)** SLF–PPC for aSC > eSC. The connectivity blobs are thresholded and cluster-level corrected using FDR for multiple comparison at *p* = 0.05. Color bar coding: violet represents an increased (positive) and reduced (negative) in functional connectivity for the egocentric and aSC condition, respectively, whereas the hot represents an increased (positive) and reduced (negative) in functional connectivity for the aSC and eSC condition, respectively compared to one another. Precu, precuneus; SMA, somatosensory motor area; SMG, supramarginal gyrus; SFG, superior frontal gyrus; MFG, middle frontal gyrus; pITG, posterior division of inferior temporal gyrus; PCC, posterior cingulate cortex; SFG, superior frontal gyrus; sLOC, superior division of lateral occipital cortex; PO, parietal operculum; MedFC, medial prefrontal cortex; PaCiG, paracingulate gyrus; PoCG, postcentyral gyrus; TPJ, temporoparietal junction.

There were associations between the FA values of the SLF and DAN under the influence of the aSC and eSC task effects (Figure 5, Table 3). Compared to the older participants, the FA values of the SLF among the younger participants was significantly associated with the connectivity between the right FEF and SFG [T(47) = 4.37, *p* < 0.001] and right FEF and Cereb T(47) = 5.26, *p* < 0.001), and significantly and negatively associated with the connectivity between the right FEF and precuneus [T(47) = −4.34, *p* < 0.001], right FEF and aSMG [T(47) = −4.91, *p* < 0.001], and right FEF and SMA T(47) = −5.89, *p* < 0.001] for the aSC > eSC contrast. Moreover, for the aSC condition, the FA values for the younger participants showed significantly and negatively association with the connectivity between the right IPS and subcallosal cortex [SUbCal: T(47) = −5.46, *p* < 0.001], right IPS and MFG [T(47) = −4.30, *p* < 0.01], and right IPS and pITG [T(47) = −4.80, *p* < 0.01].

**Table 3:** Summary on the results for the seeds constructed within the DAN and FPN for the young_SLF > OLD_SLF as the between-subject contrast, aSC > eSC as the between-condition contrast.

**Seed**	**Functional connectivity** **region**	**K_E_**	**Hemi**	**Coordinates**			***P*_FDR_**	**Peak** **t**
				***x***	***y***	***z***		
FEF	Precuneus	1,567	R	0	−60	50	<0.001	−4.34
	Superior frontal gyrus	1,466	R	4	64	28	<0.001	4.37
	Anterior supramarginal gyrus	1,351	L	−46	−30	36	<0.001	−4.91
	Cerebellum	1,257	R	22	−84	−34	<0.001	5.26
	Somatosensory association area	966	L	−16	8	48	<0.001	−5.89
IPS	Subcallosal cortex	2,062	R	20	46	−12	<0.001	−5.46
	Middle frontal gyrus	751	R	34	14	46	0.01	−4.30
	Posterior inferior temporal gyrus	739	R	52	−18	−24	0.01	−4.80
LPFC	Posterior cingulate cortex	3,030	R	2	−46	24	<0.001	−5.03
	Superior lateral occipital cortex	696	L	−54	−66	16	0.01	−4.14
	Insular cortex	616	R	38	−14	0	0.01	4.39
	Superior frontal gyrus	464	R	−6	62	36	0.04	−4.15
	Cerebellum	456	L	−24	−84	−30	0.04	−5.04
PPC	Medial frontal cortex	1,883	L	−18	52	−22	<0.001	4.99
	Para-cingulate gyrus	1,359	R	16	48	16	<0.001	−5.49
	Parietal operculum	1,181	R	62	−20	26	<0.001	−4.62
	Postcentral gyrus	653	L	−46	−16	30	0.02	−4.65
	Temporoocipital area	578	R	22	−30	20	0.03	4.49

There were associations between the FA values in the SLF and FPN under the influence of the aSC and eSC task effects (Figure 5, Table 3). Compared to the older participants, the FA values of the SLF for the younger participants was significantly associated with the connectivity between the right LPFC and IC [T(47) = 4.39, *p* < 0.01], and significantly and negatively associated with the connectivity between the right LPFC and PCC [T(47) = −5.03, *p* < 0.001], right LPFC and sLOC [T(47) = −4.14, *p* < 0.01], right LPFC and SFG [T(47) = −4.15, *p* < 0.04], and right LPFC and cerebellum (49) = −5.04, *p* < 0.04] for the contrast between aSC > 33 eSC. Moreover, for the aSC condition, the FA values for the younger participants showed significant association with the connectivity between the right PPC and medial frontal cortex [T(47) = 4.99, *p* < 0.001] and right PPC and TPJ [T(47) = 4.49, *p* < 0.03], negatively associated with connectivity between right PPC and PaCiG [T(47) = −5.49, *p* < 0.001], right PPC and PO [T(47) = −4.62, *p* < 0.001], and right PPC and PoCG [T(47) = −4.65, *p* < 0.02].

**Figure 6:**
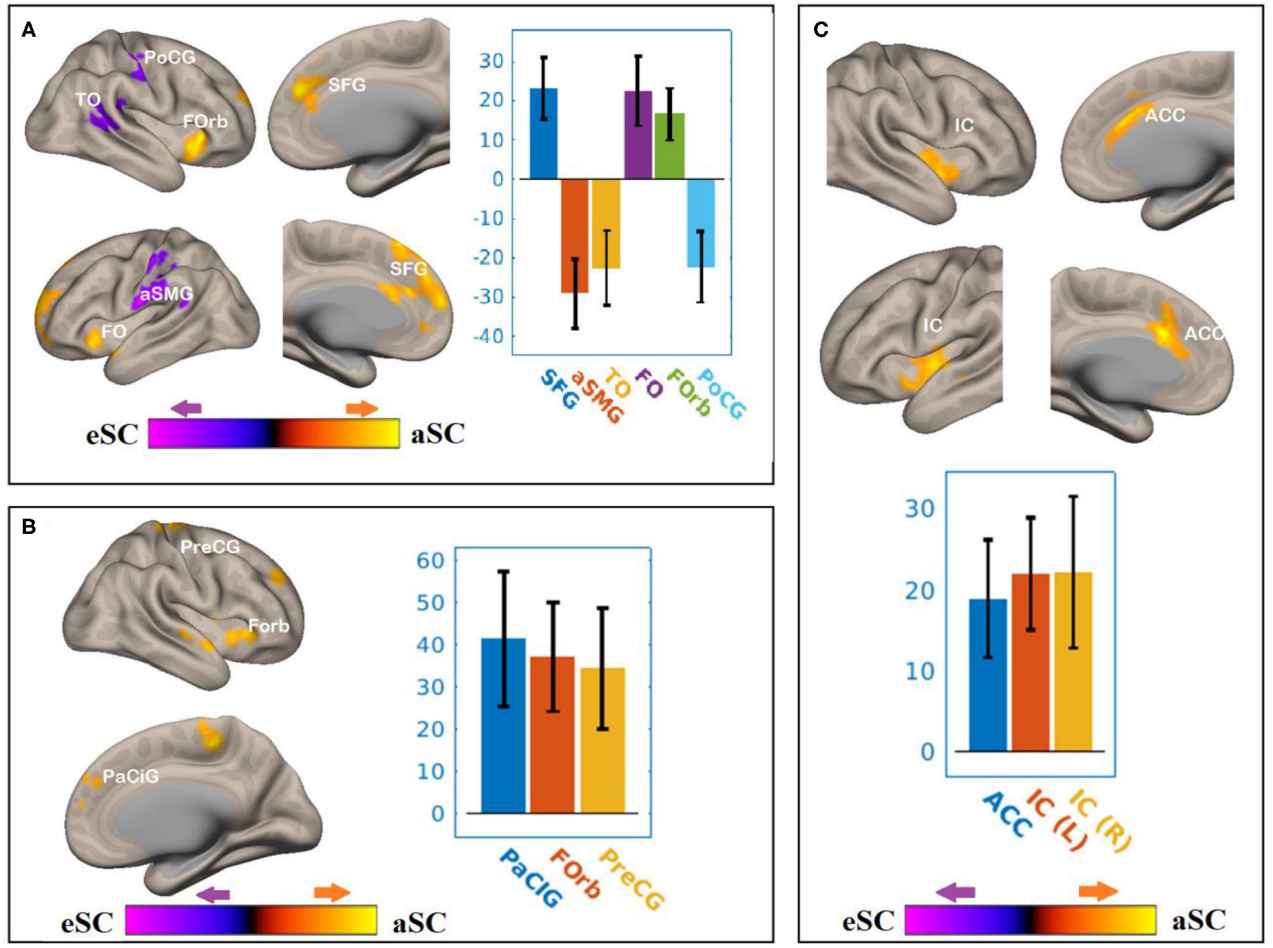
The differences between the younger and older participants in the FPN and DAN connectivity in association with the FA values of the SCR under the influence of the allocentric and egocentric task effects. **(A)** SCR–FEF for aSC > eSC. **(B)** SCR–IPS for aSC > eSC. **(C)** SCR–LPFC for aSC > eSC. The connectivity blobs are thresholded and cluster-level corrected using FDR for multiple comparison at *p* = 0.05. Color bar coding: violet represents an increased (positive) and reduced (negative) in functional connectivity for the egocentric and allocentric condition, respectively, whereas the hot represents an increased (positive) and reduced (negative) in functional connectivity for the allocentric and egocentric condition, respectively. TO, temporo-occipital; PoCG, post central gyrus; Forb, Fronto orbital cortex; SFG, superior frontal gyrus; FO, frontal operculum; aSMG, anterior division of supramarginal gyrus; PaCIG, para-cingulate gyrus; IC, inter-calarine cortex; IC, insular cortex.

There were associations between the FA values of the SLF and DAN under the influence of the aSC and eSC task effects (Figure 6, Table 4). Compared to the older participants, the FA values of the SCR among the younger participants were positively associated with the connectivity between the right FEF and SFG [T(47) = 5.06, *p* < 0.001], right FEF and FO [T(47) = 4.24, *p* < 0.02], right FEF and forb [T(47) = 4.16, *p* < 0.02], and significantly and negatively associated with the connectivity between the right FEF and TO [T(47) = −3.98, *p* < 0.001], right FEF and aSMG [T(47) = −5.59, *p* < 0.001], right FEF and PoCG [T(47) = −4.15, *p* < 0.04] for the aSC > eSC. Moreover, for the allocentric condition, the FA values for the younger participants showed significant association with the connectivity between the right IPS and PaCig [T(47) = 4.55, *p* < 0.01], right IPS and Fob [T(47) = 4.85, *p* < 0.01], right IPS and PrecG [T(47) = 4.02, *p* < 0.01].

**Table 4:** Summary on the results based on the seeds constructed within the DAN and FPN for the young_SCR > old_SCR as the between-subject contrast, and the aSC > eSC as the between-condition contrast.

**Seed**	**Functional connectivity** **region**	**K_E_**	**Hemi**	**Coordinates**			***P*_FDR_**	**Peak** **t**
				***x***	***y***	***z***		
FEF	Superior frontal gyrus	2,479	L	2	44	26	<0.001	5.06
	Anterior supramarginal gyrus	1,339	L	−64	−14	22	<0.001	−5.59
	Temporooccipital gyrus	756	R	50	−38	18	<0.001	−3.98
	Fronto oribital cortex	556	L	−20	22	−4	0.02	4.24
	Fronto orbital cortex	523	R	28	28	−2	0.02	4.16
	Post central gyrus	433	R	58	−18	50	0.04	−4.15
IPS	Para-cingulate gyrus	946	R	0	48	16	0.01	4.55
	Fronto oribital cortex	839	R	34	36	0	0.01	4.85
	Precentral gyrus	748	L	−4	−36	56	0.01	4.02
LPFC	Anterior cingulate	1,091	L	−2	20	32	<0.001	5.58
	Insular cortex	848	L	−60	−10	4	<0.001	4.33
	Insular cortex	557	R	48	−10	0	0.02	4.10

There were associations between the FA values of the SCR and DAN under the influence of the allocentric and egocentric task effects (Figure 6, Table 4). Compared to older participants, the FA values of the SCR among the younger participants were significantly associated with the connectivity between the right FEF and SFG [T(47) = 5.06, *p* < 0.001], right FEF and FO [T(47) = 4.24, *p* < 0.02], right FEF and forb [T(47) = 4.16, *p* < 0.02], and significantly and negatively associated with the connectivity between the right FEF and TO [T(47) = −3.98, *p* < 0.001], right FEF and aSMG [T(47) = −5.59, *p* < 0.001], right FEF and PoCG [T(47) = −4.15, *p* < 0.04] for the aSC > eSC contrast. Moreover, for the allocentric condition, the FA values for the younger participants showed significant association with the connectivity between the right IPS and PaCig [T(47) = 4.55, *p* < 0.01], right IPS and Fob [T(47) = 4.85, *p* < 0.01], right IPS and PrecG [T(47) = 4.02, *p* < 0.01].

## Discussion

The gPPI analysis examined the difference between aSC and eSC task-dependent brain network organizations of the DAN and FPAN in aging and delineated its association to the white-matter tracts of the PCR, SCR, and SLF. Efficient modulation of both allocentric and egocentric spatial coding in FPAN requires structure–function interaction. Allocentric task-modulated connectivity of the FPN and DAN with the temporal lobe was influenced by the aging differences of the white-matter tracts of the PCR and SCR, respectively. On the other hand, aging difference of the SLF mainly influenced the egocentric-task modulated connections of the DAN and FPN with frontal regions and posterior cingulate cortex. This study suggested that functional connections of the FPAN with near and far task-relevant nodes vary significantly with age and conditions. Overall, the results showed variability in the magnitude and direction of connectivity changes in association with different white-matter ROIs in to response to aSC and eSC along DAN and FPN. Covarying with aging difference in FA, aSC task-modulated connectivity changes of FEF brought negative connectivity association with sLOC, parietal regions (precuneus, SMG), and frontal regions (SMA, SFG, and paracingulate gyrus) and an increase in connectivity mainly in frontal regions (SFG, fronto-orbital cortex).

Frontal eye-fields connectivity changes could be interpreted in two equally appealing ways that efficient aSC processing in younger adults may have required lesser resources compared to older adults along the interpretation of neural efficiency (66, 67), and that the difference between aSC and eSC processing may have attributed to the nature of FEF connection to near (e.g., SFG and paracingulate gyrus) and far (e.g., sLOC and precuenus) brain regions. FEF connection tends to facilitate an eSC processing in sLOC, precuneus, SMG, and paracingulate gyrus than it facilitates for the aSC. These evidences are consistent to previous studies highlighted the role of FEF in processing top–down content of eSC (1, 7, 8). Ptak and Schnider (18) suggested that FEF holds neurons to encode egocentric associated action template. In addition, using aSC and eSC task requiring top–down attention allocation, the neural pathway of the FEF to IPS was revealed to be unique to the egocentric spatial coding (2), suggesting that compared to younger adults, the slower RT and lesser FC in FEF among older adults may have been accounted for by the difference in connectivity within the DAN (IPS, FEF). However, the FC results shown that a strong preference of FEF during eSC over the IPS was observed. This also tends to supports single cell recording study showing that parietal (PPC) and frontal (FEF) neurons detect target locations at a different pace across paradigms in visual attention in that FEF encode targets requiring top–down allocation earlier than PPC neurons and PPC neurons encodes targets in bottom–up attention processing earlier than the FEF neurons (68).

Allocentric task-modulated connectivity of FEF was also observed with anterior division of temporo-fusiform cortex, consistent to the mainstream hypothesis that aSC subserved by the ventral stream and that the aSC tend to demand working memory resources, which involves the temporal lobe (14–16). From the hierarchical multiple regression results, among older adults, it was shown that the account variance attributed by FA in PCR to the aSC activities of IPL was above and beyond the other WM tracts. Using PCR as a covariate, negative aSC-modulated connectivity exists between FEF and anterior temporal fusiform cortex and FEF and central opercular cortex was shown. These two regions are thought to engage in memory-guided visuospatial attention tasks (69), and thus PCR might mediate between these two neural areas in aSC.

Regarding the FPAN mask in association with WM tract ROIs on aSC and eSC task-modulated FC, the results showed that, compared to eSC, a greater age-related decline of aSC-modulated connectivity of the LPFC with lingual gyrus, posterior STG, anterior ITG, and insular cortex was observed. The connectivity between LPFC and temporal regions is the key in maintaining attention control (41) and encodes in an aSC map (Ref.). Age-related decline of connectivity of LPFC temporal regions was strongly linked to disrupt the feedforward and feedback loop of signals projected to frontal regions for attention execution (42). The results are in agreement with neuroimaging evidence, which showed that older adults tends to rely (preserved) on egocentric orienting (43) alongside specific reduction in aSC orienting (44), which was linked to the alteration of structure–function relationship of the LPFC-MT pathway (1, 14, 46), especially when the task at hand demands visual short-term memory involvement (45). As a consequence of aging effects on the relationship of brain structure and function in orienting and reorienting, differences in the mechanisms underlying spatial representations has been reported (3). The WM nodes in corona radiata was suggested to explain the age-related decline in attention control (41), which the present evidence strongly supports.

The other key region of FPAN examined was the PPC. In association with WM tract of the PCR on aSC and eSC task-modulated FC, the results showed that, compared to eSC, a greater age-related decline of aSC modulated connectivity of the PPC with inferior LOC and anterior ITG was observed. It has been shown that the PPC is a part of the DAN maintaining visuospatial control of the primed action (70), and LOC is parts of ventral parts of the occipito-temporal cortex modulated by long-term representation of objects in the visual-field (71). This implicates that maintenance of aSC may have required both the dorsal (for action control) and ventral stream (for memory-guided object recognition on the visual field) interactions. The WM tract in PCR may have played a greater role in connecting both streams for age-related decline in aSC. Unlike eSC, aSC may have dominated by maintaining visual scene and retrieved the task rule [see (72)]. If eSC is preserved and aSC processing capacity is reduced due to aging (3) on compromise attention control areas (73, 74), then aSC-modulated connectivity of LPFC may have been the hot spot of the functional difference. The results clearly support this premise that compared to older adults, younger adults showed positive (an increase) aSC-modulated connectivity of LPFC with ACC and insular cortex. The fronto-insular cortex and ACC is thought to play a critical role in switching between task-associated rules and executive attention (74–76), suggesting that aging may have altered the switching capabilities between aSC and eSC. The alteration of aSC-modulated connectivity during aSC execution might have linked to the WM nodes of the superior corona radiata.

This study has several limitations, and readers should interpret the results with caution. First, to examine the function–structure interaction, only BOLD signal of the contrast between aSC > eSC was used. Generalization of the results must consider the task-specific differences of the connectivity for aSC > neutral and eSC > neutral. Second, to obtain the FA values, the structural ROI were defined using previous studies on behavior–FA relationships. Readers must consider the reproducibility differences between data-driven and predefined ROI quantifications. Lastly, it is unclear whether age-related differences in WM integrity represent age-related differences in spatial coding with those predefined ROI tracts, or instead are global effects occurring across as age.

## Data Availability Statement

The raw data supporting the conclusions of this article will be made available by the authors, without undue reservation.

## Ethics Statement

The studies involving human participants were reviewed and approved by Ethic Committees of the Affiliated Rehabilitation Hospital, Fujian University of Traditional Chinese Medicine and written informed consent was obtained from all participants prior to the experiment. The patients/participants provided their written informed consent to participate in this study.

## Author Contributions

AD: conceptualization, analysis, investigation, method, writing–original draft, and writing–review and editing. BC: supervision and writing–review and editing. CC: funding acquisition, conceptualization, supervision, validation, writing–original draft, and writing–review and editing. All authors contributed to the article and approved the submitted version.

## Funding

The General Research Fund of Research Grant Council of Hong Kong (151044) partially supported this study.

## Conflict of Interest

The authors declare that the research was conducted in the absence of any commercial or financial relationships that could be construed as a potential conflict of interest.

## Publisher's Note

All claims expressed in this article are solely those of the authors and do not necessarily represent those of their affiliated organizations, or those of the publisher, the editors and the reviewers. Any product that may be evaluated in this article, or claim that may be made by its manufacturer, is not guaranteed or endorsed by the publisher.
