# Corrigendum: Functional and structural architectures of allocentric and egocentric spatial coding in aging: A combined DTI and fMRI study

**DOI:** 10.3389/fneur.2022.1006645

**Published:** 2022-08-17

**Authors:** Abiot Y. Derbie, Bolton K. H. Chau, Chetwyn C. H. Chan

**Affiliations:** ^1^Applied Cognitive Neuroscience Laboratory, Department of Rehabilitation Sciences, The Hong Kong Polytechnic University, Kowloon, Hong Kong SAR, China; ^2^Department of Psychology, Bahir Dar University, Bahir Dar, Ethiopia; ^3^Department of Psychology, The Education University of Hong Kong, Tai Po, Hong Kong SAR, China

**Keywords:** FPAN, allocentric spatial coding, egocentric spatial coding, spatial representation, frame of reference, white matter integrity, functional magnetic brain imaging (fMRI)

In the published article, there was an error regarding the affiliation for Bolton K. H. Chau. Instead of “Department of Psychology, Bahir Dar University, Bahir Dar, Ethiopia” it should be “Applied Cognitive Neuroscience Laboratory, Department of Rehabilitation Sciences, The Hong Kong Polytechnic University, Kowloon, Hong Kong SAR, China.”

The authors apologize for this error and state that this does not change the scientific conclusions of the article in any way. The original article has been updated.

## Publisher's note

All claims expressed in this article are solely those of the authors and do not necessarily represent those of their affiliated organizations, or those of the publisher, the editors and the reviewers. Any product that may be evaluated in this article, or claim that may be made by its manufacturer, is not guaranteed or endorsed by the publisher.

